# The Potential Role of Growth Differentiation Factor 15 in COVID-19: A Corollary Subjective Effect or Not?

**DOI:** 10.3390/diagnostics12092051

**Published:** 2022-08-24

**Authors:** Ahmad O. Babalghith, Hayder M. Al-kuraishy, Ali I. Al-Gareeb, Michel De Waard, Jean-Marc Sabatier, Hebatallah M. Saad, Gaber El-Saber Batiha

**Affiliations:** 1 Medical Genetics Department, College of Medicine, Umm Al-Qura University, Mecca 24382, Saudi Arabia; 2 Department of Clinical Pharmacology and Medicine, College of Medicine, Al-Mustansiriya University, Baghdad P.O. Box 14022, Iraq; 3 Smartox Biotechnology, 6 rue des Platanes, 38120 Saint-Egrève, France; 4 L’institut du Thorax, INSERM, CNRS, UNIV NANTES, F-44007 Nantes, France; 5 LabEx Ion Channels, Science & Therapeutics, Université de Nice Sophia-Antipolis, F-06560 Valbonne, France; 6 Institut de Neurophysiopathologie (INP), Aix-Marseille Université, CNRS UMR 7051, Faculté des Sciences Médicales et Paramédicales, 27 Bd Jean Moulin, 13005 Marseille, France; 7 Department of Pathology, Faculty of Veterinary Medicine, Matrouh University, Mersa Matruh 51744, Egypt; 8 Department of Pharmacology and Therapeutics, Faculty of Veterinary Medicine, Damanhour University, Damanhour 22511, Egypt

**Keywords:** COVID-19, growth differentiation factor 15, hyperinflammation, exaggerated immune response, acute lung injury

## Abstract

Coronavirus disease 2019 (COVID-19) is primarily caused by various forms of severe acute respiratory syndrome coronavirus type 2 (SARS-CoV-2) variants. COVID-19 is characterized by hyperinflammation, oxidative stress, multi-organ injury (MOI)-like acute lung injury (ALI) and acute respiratory distress syndrome (ARDS). Different biomarkers are used in the assessment of COVID-19 severity including D-dimer, ferritin, lactate dehydrogenase (LDH), and hypoxia-inducible factor (HIF). Interestingly, growth differentiation factor 15 (GDF15) has recently become a potential biomarker correlated with the COVID-19 severity. Thus, this critical review aimed to determine the critical association between GDF15 and COVID-19. The perfect function of GDF15 remains not well-recognized; nevertheless, it plays a vital role in controlling cell growth, apoptosis and inflammatory activation. Furthermore, GDF15 may act as anti-inflammatory and pro-inflammatory signaling in diverse cardiovascular complications. Furthermore, the release of GDF15 is activated by various growth factors and cytokines including macrophage colony-stimulating factor (M-CSF), angiotensin II (AngII) and p53. Therefore, higher expression of GDF15 in COVID-19 might a compensatory mechanism to stabilize and counteract dysregulated inflammatory reactions. In conclusion, GDF15 is an anti-inflammatory cytokine that could be associated with the COVID-19 severity. Increased GDF15 could be a compensatory mechanism against hyperinflammation and exaggerated immune response in the COVID-19. Experimental, preclinical and large-scale clinical studies are warranted in this regard.

## 1. Introduction

Coronavirus disease 2019 (COVID-19) is primarily caused by various forms of severe acute respiratory syndrome coronavirus type 2 (SARS-CoV-2) variants [1,2]. The last SARS-CoV-2 variant was Omicron, which was declared by the World Health Organization (WHO) on 26 November 2021 and spread to more than 136 countries [3,4]. COVID-19 is characterized by hyperinflammation, oxidative stress, multi-organ injury (MOI)-like acute lung injury (ALI) and acute respiratory distress syndrome (ARDS) [5,6]. COVID-19 patients are commonly asymptomatic in most cases, reaching up to 85%. However, 15% of COVID-19 patients may present with a moderate to severe form due to progression of SARS-CoV-2 infection with development of ALI. In addition, 5% of COVID-19 patients may develop a critical form due to the development of ARDS that necessities ventilator support and mechanical ventilation [1,7].

In this state, different biomarkers are used in the assessment of COVID-19 severity including D-dimer, ferritin, lactate dehydrogenase and hypoxia-inducible factor (HIF) [8]. Of interest, growth differentiation factor 15 (GDF15) has been recently to be a potential biomarker correlated with COVID-19 severity [9]. Thus, the objective of the present critical review was to find the critical association between GDF15 and COVID-19 regarding the disease severity and clinical outcomes.

## 2. Growth Differentiation Factor 15

Growth differentiation factor 15 (GDF15) was first recognized as a macrophage inhibitory cytokine-1 (MIC) in 1990. GDF15 is belonging to the transforming growth factor-beta (TGF-β) superfamily and is regarded as a stress-response member of TGF-β [10]. GDF15 is encoded by human chromosome 19p13.11-13.2 which was cloned in 1997 through macrophage activation [11]. GDF15 is typically found in a low concentration, with the exception of the placenta which highly expresses the GDF15 during pregnancy. GDF15 is increased during pregnancy and following organ injury chiefly lung and liver [10]. Under physiological conditions, it is highly expressed by adipocytes, skeletal, smooth and cardiac muscle cells as well as macrophages [10].

The perfect function of GDF15 remains not well-recognized; nevertheless, it plays a vital role in controlling cell growth, apoptosis and inflammatory activation [12]. Consequently, GDF15 is observed as a prognostic biomarker in cancer, inflammatory diseases, and cardiovascular complications [10]. Indeed, GDF15 is overexpressed in various cancer cell types including renal, prostatic, colorectal, urothelial and melanoma [13]. GDF15 persuades weight loss via the suppression of appetite, thus neutralizing antibodies against the GDF15 may reduce cancer-mediated cachexia [14].

**Figure 1:**
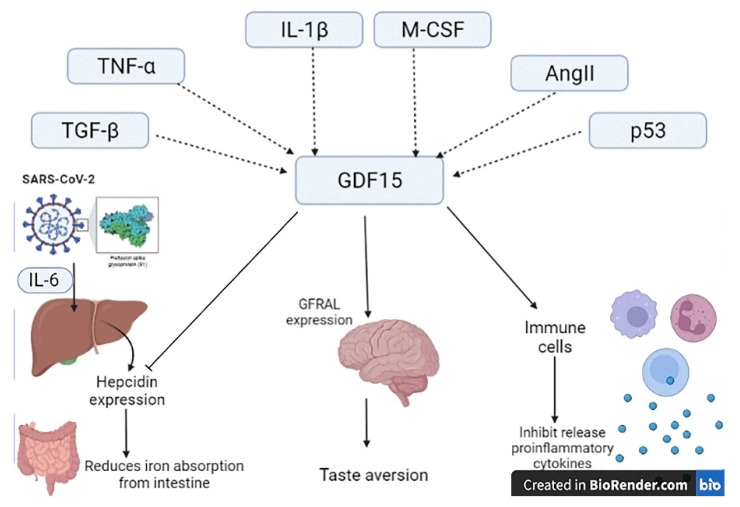
Activation of growth differentiation factor 15 (GDF15) and its action. GDF15 is activated by TGF-β (transforming growth factor-β), TNF-α (tumor necrosis factor-α), IL (interleukin)-1β, M-CSF (macrophage colony-stimulating factor), AngII (angiotensin II) and p53. GDF15 stimulates immune cells and activates glial-derived neurotrophic factor family receptor α-like (GFRAL) in the brain. In addition, GDF15 counteracts hepcidin which increased in various viral infections including SARS-CoV-2 due to the elevation of IL-6.

Furthermore, the GDF15 may act as anti-inflammatory and pro-inflammatory signaling in diverse cardiovascular complications. It has been reported that the p53 protein promotes the expression of GDF15 during inflammation and oxidative stress [15]. Furthermore, the release of GDF15 is activated by various growth factors and cytokines including TGF-β, tumor necrosis factor (TNF)-α, interleukin-1β (IL-1β), macrophage colony-stimulating factor (M-CSF), angiotensin II (AngII) and p53 (Figure 1) [16]. Furthermore, the endoplasmic reticulum stress was regarded as a key factor in the expression of macrophage GDF1 through the induction saturation of free fatty acids and unfolding of protein response [17]. These findings suggest that the expression of GDF1 is expressed by various cell types under physiological and pathological conditions.

The GDF15 was concerned with the development of different cardiometabolic disorders and cancer [18]. However, recent studies showed that the GDF15 is considered a cytokine that has an anti-inflammatory effect and increases insulin sensitivity which may decrease body weight and ameliorate the clinical outcomes in diabetic patients [18]. In healthy subjects, the higher expression of GDF15 reduces appetite and inflammation with the upgrading of insulin sensitivity. Nevertheless, in chronic metabolic and inflammatory disorders, the over-expression of GDF15 may cause desensitization of central and peripheral receptors of the GDF15 with subsequent elevation of GDF15 serum levels [15,16,18]. Moreover, the GDF15 has been reported to increase in various cardiometabolic and inflammatory disorders including heart failure and rheumatoid arthritis [19,20]. A systematic review study that included 21 clinical studies illustrated that GDF15 serum level was regarded as a novel biomarker of heart failure [19]. A prospective study involving 46 patients with rheumatoid arthritis compared to 36 matched healthy controls revealed that serum level of GDF15 was higher in rheumatoid arthritis patients as compared to the controls [20].

Indeed, the GDF15 activates a specific receptor called glial-derived neurotrophic factor (GDNF) family receptor α-like (GFRAL) which is highly expressed in the brain stem to induce taste aversion (Figure 1) [21,22]. The GFRAL receptors mediate the metabolic action of GDF15. Dysregulation in the expression and sensitivity of GFRAL receptors may be implicated in the pathogenesis of diabetes mellitus and obesity [22].

## 3. Growth Differentiation Factor 15 and Viral Infections

The GDF15 regulates host immune defense against various viral infections. Overexpression of the GDF15 in the human airway facilitates replication of rhinovirus and inflammation through inhibition of interferon gamma (INF-γ) [23]. Remarkably, the GDF15 is regarded as an inducer of sepsis tolerance through the modulation of metabolic alterations in severe septic infections [24].

Thus, the overexpression of GDF15 in the airway of cigarette smoker subjects may increase the risk of respiratory viral infections [23]. Moreover, the GDF15 serum level is increased in different respiratory disorders including pulmonary hypertension [25] and bronchopulmonary dysplasia [26]. Si et al. [27] illustrated that the GDF15 in hepatoma cells increases the infectivity of hepatitis C virus (HCV). Herein, the elevation of the GDF15 serum level may be a prognostic diagnostic biomarker for the severity of HCV infection [27]. Likewise, the upregulation of GDF15 is correlated with severity and poor clinical outcomes of hepatitis B virus (HBV) infection in patients with hepatocellular carcinoma [28]. Notably, HBV and HCV promote the expression of GDF15 with subsequent alteration of host immune response, growth, and signaling of host cells [28]. Interestingly, the GDF15 is highly expressed in males as compared to females as it is inversely correlated with testosterone level [29]. Recently, overexpression of anti-inflammatory GDF15 has been found to reduce the infectivity and severity of H5N1 [30]. Despite the GDF15 role in both viral and bacterial infections, it induces a protective effect by inducing metabolic tolerance against infection-induced inflammation [31].

In brief, there is a conflicting controversy regarding the possible role of GDF15 in various viral infections.

## 4. Growth Differentiation Factor 15 and COVID-19

It has been shown that the GDF15 serum level is correlated with the COVID-19 severity [9]. A small size prospective study involved 58 survivor COVID-19 patients compared to 8 non-survivor COVID-19 patients showed that a higher GDF15 serum level was associated with higher mortality [9]. Ahmed et al. [32] confirmed that a higher GDF15 serum level was regarded as a prognostic biomarker and correlated with COVID-19 severity. Teng et al. [33] observed that the dynamic changes in the GDF15 serum level are related and correlated with the progression of SARS-CoV-2 infection, and could be an indicator of the COVID-19 severity. Therefore, GDF15 serum level could be a possible diagnostic and prognostic biomarker in severely affected COVID-19 patients.

Of interest, GDF15 could be a promoter of COVID-19 tolerance in the early phase of SARS-CoV-2 infection to a detrimental factor in the propagation of the cytokine storm [32,34]. Thus, in virtue of the anti-inflammatory action of GDF15, recombinant GDF15 might be of therapeutic value against the SARS-CoV-2 infection-induced hyperinflammation [32,35]. A retrospective study comprised 111 COVID-19 patients compared to 20 healthy controls revealed that the GDF15 serum was correlated with critical patients, but decreased in recovered COVID-19 patients at the time of discharge [33]. Pooled analysis demonstrated that the GDF15 serum was significantly correlated with most of COVID-19 regardless of its severity [36]. However, many studies implicated the role of GDF15 in the pathogenesis and severity of COVID-19 [37,38]. A longitudinal study including patients with end-stage kidney disease with/without COVID-19 revealed that GDF15 was regarded as a novel biomarker linked with the COVID-19 severity [38].

Notably, GDF15 is induced by inflammation and oxidative stress to limit tissue injury by its anti-inflammatory effect [39]. GDF15 acts on the immune cells to inhibit the release of pro-inflammatory cytokines. Therefore, the GDF15 can attenuate abnormal immune responses and prevent the associated inflammation [39]. GDF15 deficiency provokes hepatic injury in mice through over-activation of neutrophils and T cell-induced hepatic inflammation and fibrosis [16]. Therefore, the recombinant GDF15 could be a potential therapeutic modality against alcohol-induced liver injury and fibrosis [40]. Furthermore, the GDF15 prevents lipopolysaccharide-induced ALI in mice [41]. In a similar way, GDF15 serum level had been reported to be augmented in septic patients [41], which might be a compensatory mechanism against immune dysregulation in sepsis.

Furthermore, the GDF15 was implicated in the pathogenesis of anemia by inhibiting the expression of hepcidin. However, hypoxia and anemia activate the expression and synthesis of GDF15 [42,43]. Lower hepcidin serum level is linked with COVID-19 severity and mortality [44,45]. Indeed, hepcidin reduces iron absorption from the intestines, and hepcidin expression is increased in various viral infections including SARS-CoV-2 due to the elevation of IL-6 [46]. However, most studies revealed that hepcidin serum level was reduced in severely affected COVID-19 patients due to the similarity between SARS-CoV-2 proteins and hepcidin [46,47]. For this reason, GDF15 could be increased to counteract hepcidin molecular mimicry of SARS-CoV-2 proteins (Figure 1).

These observations and studies highlighted that GDF15 serum level was increased in COVID-19 and correlated with its severity. Nonetheless, these studies did not find the causal relationship between GDF15 and COVID-19, and how and why it increased.

## 5. Growth Differentiation Factor 15 and Inflammatory Pathways in COVID-19

**Figure 2:**
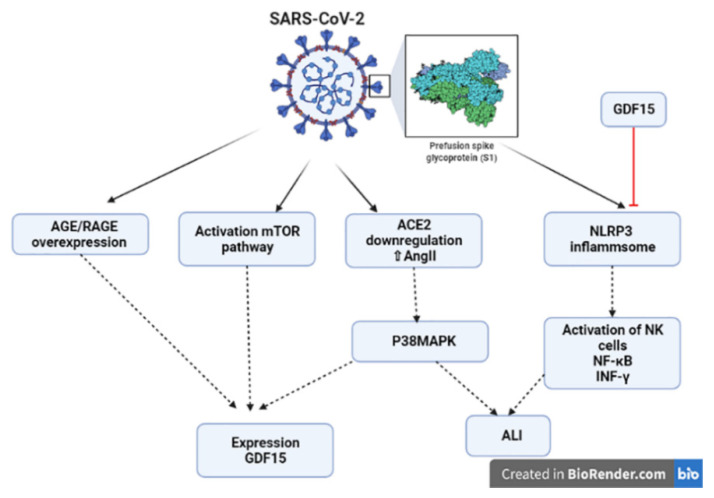
Growth differentiation factor 15 (GDF 15) and its pro-inflammatory and anti-inflammatory signaling in COVID-19. mTOR (rapamycin) pathway, ACE2 (angiotensin-converting enzyme 2), AngII (angiotensin II), p38 mitogen-activated protein kinase (p38MAPK), ALI (acute lung injury), NK cells (natural killer cells), nuclear factor kappa B (NF-κB) and interferon-gamma (INF-γ).

Many inflammatory signaling pathways are concerned with the pathogenesis of SARS-CoV-2 infection. The nod-like receptor pyrin 3 receptor (NLRP3) inflammasome is implicated in the activation of natural killer cells and the nuclear factor kappa B (NF-κB) signaling pathway with the release of INF-γ [48]. Suppression of NLRP3 inflammasome may decrease exaggerated immune response-induced organ injury [48]. Over-activated GDF15 in experimental diabetes inhibits the progression of inflammatory reaction through inhibition of NLRP3 inflammasome (Figure 2) [49]. In addition, the GDF15 attenuates the progression of ALI by inhibiting sirtuin (SIRT) and NLRP3 inflammasome in the animal model study [41].

Notably, the NF-κB signaling pathway is extremely activated in the SARS-CoV-2 infection by ORF7a SARS-CoV-2 viral protein with the succeeding expression of pro-inflammatory cytokines [50]. Thus, NF-κB inhibitors have an immense role in the reduction in SARS-CoV-2 infection by lessening the expression of inflammatory cytokines [50]. The exaggerated immune response may increase the expression of GDF15 through NF-κB signaling-dependent pathway [51].

Certainly, the p38 mitogen-activated protein kinase (p38MAPK) is a pro-inflammatory pathway linked with the development of ALI and acute cardiac injury in COVID-19 [52]. Overactivation of the p38MAPK in COVID-19 could be due to the down-regulation of angiotensin-converting enzyme 2 (ACE2) and an increase in AngII level (Figure 2). In addition, the SARS-CoV-2 can directly activate the p38MAPK signaling pathway causing endothelial dysfunction, vasoconstriction and thrombosis [52]. The GDF15 is activated by p38MAPK in chondrogenesis [53].

Moreover, the mechanistic target of the rapamycin (mTOR) pathway is the innermost regulator of cell growth, proliferation, metabolism and survival [54]. This pathway is a member of the protein kinases that senses both extracellular and intracellular regulatory signals to manage autophagy, the expression of inflammatory genes and organelle biogenesis [54]. It has been shown that the mTOR pathway is activated during the SARS-CoV-2 infection, and involved in the transcription and mRNA translation of the SARS-CoV-2 particles [55]. Of interest, the mTOR pathway and associated pro-inflammatory cytokines induce the expression of GDF15 to be increased in the different inflammatory disorders (Figure 2) [56].

Advanced glycation endproducts (AGE) and receptors for AGE (RAGE) are implicated in the pathogenies of SARS-CoV-2 infection; however, the soluble RAGE (sRAGE) has a protective effect against the COVID-19 severity [57]. Indeed, overexpression of the AGE/RAGE is associated with COVID-19 severity and mortality [57]. Different studies confirmed that the overexpression of AGE/RAGE is correlated with the induction of the release of GDF15 (Figure 2) [58,59].

Of interest, the forkhead box O (FoxO) is a transcription factor that plays an important role in cell homeostasis through the regulation of apoptosis, oxidative stress and maturation of lymphocytes [60]. FoxO has anti-inflammatory effects, so its activators may reduce COVID-19 severity [61]. FoxO anti-inflammatory effect may decrease disease severity and aging through modulation of inflammatory milieu and cell homeostasis [62]. FoxO modulates the expression of GDF15 in rats with ischemic/reperfusion injury [63].

Interestingly, the GDF15 is highly activated in diabetic patients due to metabolic derangement and over-activated AngII [18]. Higher expression of the GDF15 in diabetes mellitus plays a crucial role in the attenuation of inflammatory and metabolic complications [18]. Notably, AngII is highly activated due to the downregulation of ACE2 by SARS-CoV-2 leading to induction of ALI/ARDS and thrombotic complications [64].

Furthermore, hypoxia-inducible factor 1 (HIF-1) is provoked in COVID-19 that may have beneficial and detrimental effects [65]. Notably, HIF-1 induces the expression of GDF15 in cancer metastasis [66]. Thus, higher serum levels of GDF15 in severely affected COVID-19 patients mirror hypoxic state.

These observations suggest that triggered inflammatory signaling together with stimulated AngII and HIF-1 which are involved in the pathogenesis of SARS-CoV-2 could be the underlying causes of high GDF15 in the COVID-19. Therefore, the higher expression of GDF15 in COVID-19 might a compensatory mechanism to stabilize and counteract dysregulated inflammatory reactions through the inhibition of inflammatory signaling pathways and augmentation of anti-inflammatory pathways.

## 6. GDF15 in Comparison with Other COVID-19 Biomarkers

In comparison with well-known biomarkers of COVID-19, the GDF15 is increased in parallel with other inflammatory biomarkers in COVID-19 patients. An observational study involving 66 hospitalized COVID-19 patients demonstrated that both GDF15 and calprotectin serum levels were increased and correlated with disease severity and mortality [9]. GDF15 has a similar prognostic value to that of calprotectin in the prediction of COVID-19 complications and severity [9]. A retrospective study that included 440 COVID-19 patients showed that the GDF15 serum level was increased and positively correlated with C reactive protein (CRP), IL-6 and IL-8 in severely affected patients [33]. Gisby et al. [38] found that the GDF15 serum level together with IL-8 was effective in monitoring COVID-19 in patients with end-stage kidney disease. Furthermore, a case–control study comprising 80 patients with moderate to severe COVID-19 showed that the GDF15 serum level was increased together with increasing levels of galectin-9 and C3a in severely affected patients [67]. The rising of GDF15, galectin-9 and C3a in COVID-19 patients reflect intestinal tight junction dysfunction with translocation of intestinal microbes into the circulation with the development of systemic inflammation [67]. Interestingly, Myhre and colleagues confirmed in a prospective observational study that the GDF15 offers a prognostic biomarker superior to other inflammatory biomarkers in unselected hospitalized COVID-19 patients [68]. GDF15 in severely affected COVID-19 patients is more specific than IL-6, CRP, ferritin and D-dimer in detecting the early stage of COVID-19 severity and admission to the intensive care unit (ICU) [68]. A prospective study involved 135 COVID-19 patients, 35 (28%) of them were admitted to ICU and 97 (79%) had higher GDF15 baseline level. GDF15 serum level was highly sensitive and specific, correlated with ICU admission of severely affected patients (0.78, 95%CI = 0.07–0.86) [68].

These findings pointed out that GDF15 is regarded as a noteworthy diagnostic/prognostic biomarker in detecting COVID-19 severity and complications.

## 7. Modulation Release of GDF15

Of interest, metformin stimulates the release of GDF15 [69] that have an imperative effect in treating SARS-CoV-2 infection [70]. Metformin inhibits the interaction between SARS-CoV-2 and ACE2 by inhibiting the release of pro-inflammatory cytokines [70]. Kleinert et al. [29] study illustrated that physical exercise stimulates and improves the release of GDF15. In turn, regular physical exercise improves immune tolerance to COVID-19 [71]. Herein, metformin and physical exercise could prevent immune dysregulation and hyperinflammation through the modulation expression of GDF15 patients. Similarly, colchicine increases the expression and the release of hepatic GDF15 [32]. Different studies confirmed that colchicine decreased COVID-19 severity via the regulation of immune response to SARS-CoV-2 infection [72,73,74]. The case series by Al-Kuraishy et al. [73] including COVID-19 patients treated by sequential use of doxycycline in the first week and colchicine in the second week led to significant improvement in clinical outcomes. Unfortunately, the GDF15 serum levels were not measured in our previous study. A systematic review and meta-analysis by Yasmin et al. [75] involved randomized clinical trials regarding the safety and effectiveness of colchicine in COVID-19 patients demonstrating that colchicine was effective and safe in the management of COVID-19. However, another systematic review illustrated that colchicine was infective in reducing mortality of hospitalized COVID-19 patients [76].

## 8. Mitochondrial Dysfunction and GDF15 in COVID-19

Mitochondria are organelles that regulate different cellular processes. Mitochondrial stress is generated due to defects in the transport of electron chains with impairment of mitochondrial proteostasis [77]. Mitochondrial stress and dysfunction are developed in response to abnormal immune responses and metabolic disturbances as in sepsis [77]. Under the effect of mitochondrial stress and dysfunction, various genes in cell survival are transcriptionally activated [78]. Mitochondrial stress triggers the release of various secretory proteins from cells such as the GDF15 and fibroblast growth factor 2, enabling cells with mitochondrial dysfunction to communicate with distant and neighboring cells to modulate the cell metabolism and energy [77]. Montero et al. [79] revealed that the GDF15 serum level was increased in children with inherited mitochondrial diseases.

Notably, severe SARS-CoV-2 infection is associated with the development of mitochondrial stress and dysfunction due to the exaggerated immune response and hyperinflammation [80,81]. It has been shown that the SARS-CoV-2 infection is linked with noteworthy alteration of mitochondrial dynamics with subsequent development of oxidative stress, the release of the pro-inflammatory cytokines and propagation of the cytokine storm [80]. The regulation of mitochondrial membrane potential by fucoidan may prevent the development of mitochondrial dysfunction in COVID-19 patients [81]. A study conducted by De la Cruz-Enríquez et al. [82] showed that inflammation/oxidative markers were correlated with mitochondrial dysfunction in the leukocytes of COVID-19 patients. In turn, mitochondrial dysfunction promotes the propagation of oxidative stress and hyperinflammation with subsequent development of endothelial-alveolar injury [82].

Therefore, these verdicts suggest that the increasing levels of GDF15 might be due to the development of mitochondrial stress and dysfunction in severely affected COVID-19 patients.

## 9. Thrombosis and GDF15 in COVID-19

Endothelial dysfunction, oxidative stress and inflammatory disorders in the SARS-CoV-2 infection may lead to thrombotic events, the hallmark of COVID-19 [83]. The underlying causes of thromboembolic disorders in COVID-19 are due to different mechanisms including platelet activation, stimulation of clotting factors, inhibition of the endogenous anticoagulant system and fibrinolytic pathways [84]. Thromboembolic disorders in COVID-19 promote the propagation of pulmonary embolism, deep vein thrombosis and disseminated intravascular coagulopathy (DIC) [85]. It has been illustrated that COVID-19 and anti-SARS-CoV-2 vaccines are linked with a high thrombotic milieu [83,84,85]. Mosleh and colleagues [86] showed that endothelial dysfunction and endothelitis in COVID-19 patients increase the risk for the development of thrombosis. A systematic review revealed that the SARS-CoV-2 infection increases the risk of stent thrombosis in COVID-19 patients with acute coronary syndrome [83]. A meta-analysis and systematic review pointed out that venous thromboembolism was higher in hospitalized COVID-19 patients despite the use of thromboprophylaxis [87], suggesting a prominent heterogeneity of thrombosis in COVID-19.

On the other hand, the GDF15 is regarded as a prognostic biomarker of pulmonary embolism in patients with cardiovascular diseases [88]. A prospective cohort study involved 123 patients with acute pulmonary embolism revealed that the GDF15 serum level was higher and correlated with 30-day mortality [88]. In addition, there is evidence proposed that the GDF15 serum level appears to be linked with stroke in patients with atrial fibrillation [89]. An observational study included 894 patients with atrial fibrillation with or without left atrial thrombus revealed that GDF15 serum level was higher in patients with atrial thrombus compared to patients with atrial fibrillation without atrial thrombus [89]. Inflammatory reactions induce thrombosis and release GDF15 from activated macrophages [90]. However, the GDF15 knockout mice experience accelerated thrombosis compared to wild-type mice [91]. Furthermore, in vitro study demonstrated that GDF15 had the ability to inhibit platelet aggregation [91]. Thus, GDF15 might not be the putative cause of thrombosis but as a compensatory mechanism against the development of thromboembolic disorders in various cardiovascular complications [90].

In severely affected COVID-19 patients with ARDS at ICU, the anti-inflammatory IL-10 and GDF15 were increased, positively and negatively correlated with pro-inflammatory IL-6 and lymphopenia, respectively [37]. Therefore, the elevation of GDF15 in critical COVID-19 patients mirrors immunothrombotic events.

Taken together, in virtue of its anti-inflammatory effects, GDF15 may inhibit the propagation of cytokine storm in COVID-19 patients through modulation of the immune-inflammatory response and attenuation of the exaggerated immune response [32,92]. Furthermore, the activation of inflammatory signaling pathways such as NLRP3 inflammasome and NF-κB are associated with the development of cytokine storm [93]. Therefore, increasing GDF15 levels in severely affected patients could be countercurrent mechanisms to damping hyperinflammation in the cytokine storm.

The present perspective had several limitations including scarcity of clinical studies and serial measurement of the GDF15 in the initial and late phases of COVID-19 patients were not reported. However, this review—unlike other studies which implicate GDF15 in the pathogenesis and severity of COVID-19,—confirmed that the increase in GDF15 in COVID-19 could be a compensatory mechanism against hyperinflammation and exaggerated immune response.

## 10. Gastrointestinal Injury and GDF15 in COVID-19

GDF15 in disease state is highly expressed in different parts of the gastrointestinal tract (GIT) including stomach, colon, bile duct and liver. Expression of GDF15 in the liver rapidly occurs following acute liver injury independent of p53 and TNF-α pathways [94]. Furthermore, GDF15 expression is also induced following common bile injury. It has been reported that Northern blot analysis of hepatic mRNA from patients with cirrhosis and sclerosing cholangitis demonstrated a significant expression of GDF15 [95]. Lee et al. [96] illustrated that GDF15 predicts the severity of chronic liver diseases. A case–control study included 145 patients with chronic liver diseases compared to 101 matched healthy control subjects and showed that GDF15 serum level was higher in severely affected disease patients [96]. These findings suggest that GDF15 might be a possible biomarker of GIT injury.

On the other hand, COVID-19 is commonly associated with GIT injury and acute hepatic damage due to the higher expression of ACE2 [97]. Indeed, ischemic-reperfusion injury, cytokine storm, oxidative stress and drug-induced injury could be the potential mechanisms for development of GIT injury in COVID-19 [97]. GDF15 serum level is increased in COVID-19 patients with extra-pulmonary manifestations including GIT injury and acute hepatic damage [9]. A retrospective study comprising 2623 confirmed COVID-19 patients with acute hepatic injury revealed that low albumin serum level and high GDF15 serum level are correlated with COVID-19 severity and death [98]. As mentioned above, GDF15 serum level increased in parallel with calprotectin a biomarker of GIT injury in critically affected COVID-19 patients [9]. The underlying mechanism for increasing GDF15 serum level in COVID-19 patients with GIT injury and/or acute hepatic damage is due to hyperinflammation, oxidative stress and exaggeration of inflammatory signaling pathways [48,51,53].

These verdicts proposed that GDF15 could be a diagnostic and prognostic biomarker of GIT injury in COVID-19.

The potential role of GDF15 in COVID-19 is summarized in Table 1.

## 11. Conclusions

COVID-19 is characterized by hyperinflammation, oxidative stress, MOI-like ALI and ARDS. COVID-19 is associated with hyperinflammation and exaggerated immune response due to the activation of the inflammatory signaling pathway. GDF15 is an anti-inflammatory cytokine and increased GDF15 could be a compensatory mechanism against hyperinflammation and exaggerated immune response in COVID-19 so that it acts as a pathogenic marker. Of interest, GDF15 serum level may reflect the underlying hyperinflammation and associated tissue injury including pulmonary and extra-pulmonary complications. Furthermore, GDF15 serum level may predict COVID-19 severity and mortality. Therefore, GDF15 is regarded as a diagnostic and prognostic biomarker in COVID-19 patients. Experimental, preclinical and large-scale clinical studies are warranted in this regard to verify the precise role of GDF15 in COVID-19 regarding immunothrombosis.s

## Figures and Tables

**Table 1:** The potential role of GDF15 in COVID-19.

Ref.	Study Type	Findings
de Guadiana et al. [9]	Prospective study	Higher GDF15 serum level was associated with higher mortality
Ahmed et al. [32]	Review study	Higher GDF15 serum level was regarded as a prognostic biomarker and correlated with COVID-19 severity.
Teng et al. [33]	Retrospective study	Higher GDF15 serum is an indicator of the COVID-19 severity.
Lippi and Henry [36]	Pooled analysis study	The GDF15 serum was significantly correlated with most of COVID-19 regardless of its severity.
Notz et al. [37]	Observational pilot study	The GDF15 is implicated in the pathogenesis and severity of COVID-19.
Gisby et al. [38]	Longitudinal proteomic study	The GDF15 serum level is correlated with COVID-19 severity
Rochette et al. [39]	Review study	The GDF15 can attenuate abnormal immune responses and prevent the associated inflammation in COVID-19.
Giron et al. [67]	A case–control study	The GDF15 serum level was increased together with increasing levels of galectin-9 and C3a in severely affected COVID-19 patients.
Myhre et al. [68]	A prospective observational study	The GDF15 offers a prognostic biomarker superior to other inflammatory biomarkers in unselected hospitalized COVID-19 patients.
Notz et al. [37]	Observational pilot study	In severely affected COVID-19 patients with ARDS at ICU, the anti-inflammatory IL-10 and GDF15 were increased, positively and negatively correlated with pro-inflammatory IL-6 and lymphopenia, respectively.
Huang et al. [98]	A retrospective study	The High GDF15 serum level is correlated with COVID-19-induced acute hepatic injury

## Data Availability

Not applicable.
